# Dr. Parkinson's Essay on the Efficacy of Alcohol

**Published:** 1810-08

**Authors:** T. Parkinson

**Affiliations:** Leicester


### *To the Editors of the Medical and Physical Journal.*

Gentlemen,

I Beg leave to offer to your consideration the following Remarks on the superior efficacy of Alcohol, as a topical application, where the intention is to remove local inflammation by resolution or dispersion, and particularly that which arises from burning or scalding.

This Essay is the result of much practical observation, and might be supported by numberless cases; but as the subject has already been, in a short way, before the public, and some cases then presented, I shall confine myself within as narrow limits as possible; and should you think it, in this state, deserving of a place in your Medical Journal, I beg you will introduce it in your next.

I am, &c.

T. PARKINSON.

*Leicester, June 13, 1810.*

*A Chemico*

*A Chemico Medical Essay on the Efficacy of Alcohol, evaporated from Surfaces injured by Burning or Scalding.* By T. PARKINSON, M. D.

Several years ago, I published a small paper in the Medical Journal, on the Efficacy of Spirit of Wine, evaporated from surfaces injured by burning or scalding, and since that time, a great number of cases have occurred to me, which fully confirm the opinion I then advanced.—From a persuasion that it may be extended as a remedy in inflammatory diseases, intended to be removed by resolution, I now resume the subject, with a view to explain the modus agendi in such a manner, as will lead to the most favourable mode of application.

Spirit of wine, or alcohol, has been highly esteemed by the most eminent in the practice of Physic and Surgery, in cases of burning and scalding, under the professed title of an astringent; but is now superseded by the more popular preparations of lead, cold lotions, emollients, and oily liniments.

Before I proceed further, it will be needful for me to inquire, whether inflammation is to be considered a simple uncompounded property, or a compound one.

The symptoms and common causes of inflammation are so well defined and so generally understood, as not to need any notice here; excepting, that it is active inflammation only, to which the evaporation of alcohol is directed; and to divide it into phlegmonous and erysipelatous; the former of which terminates either by resolution, suppuration, or gangrene; the latter either by resolution or gangrene. This consideration must be remembered through the whole of the following observations; and I would particularly notice, that one of the prevailing causes of inflammation is the direct imposition of heat, or the contrary, the abstraction of heat.

I shall, in future, speak of heat under the appropriate term caloric, considering it one of the component parts of life, so far as relates to health, disease, and death.

In health, a relative quantity of caloric, capable, however, of considerable variation, is one of the component parts; beyond the limits of this variation, disease is produced, admitting also of considerable variation; but, beyond these limits death follows.

What quantity is required to sustain life, or what quantity is destructive of it, is not so much our present consideration, as there are limited boundaries, within which, health will be supported; others, within which, disease will will be produced; and others, beyond which, life will be destroyed.

In no condition, however, can the living fibre endure the heat of boiling water, for any length of time, without being decomposed and destroyed by it; and, may it not, with equal propriety, be said that, in no condition can the living fibre endure a degree of cold, correspondent to the heat of boiling water, without being decomposed and destroyed by it?

Caloric enters into the composition of all bodies, whether animate or inanimate; and is more or less fixed, according to the structure and peculiar properties of each individual body.

Some substances will retain their integrity, and not be perceptibly changed by the greatest abstraction that art can effect; and are, with difficulty, deranged by the imposition of caloric; whilst others assume new appearances and properties by either a slight imposition or a slight abstraction; a more striking instance need not be adduced than the form that metals exhibit, either fluid or solid, according to the quantity of caloric united with them; and more particularly that of quicksilver becoming solid, and even malleable under a degree of abstraction, of which it is capable.

Metals, like other insensible bodies, have not the power of retaining more than their natural or component quantity of caloric any longer than an excess is communicated to them, but presently part with it, until they return to their original temperature, and regain their common appearances and peculiarities: this must, however, be limited to the degree of excess of caloric, that each individual metal is capable of suffering without decomposition.

Earthy and stony substances are also composed partly of caloric, and are subject to changes equally manifest, though not altogether similar to those that happen to metals. Some, however, admit of great excess without any material alteration, whilst others are decomposed by a slight imposition; an instance is notable enough in limestone, which parts with its carbonic gas by the imposition of caloric, and though it was a mild and almost inert substance before, it now becomes active and caustic.

It seems, however, still to prefer carbonic gas, as it will take it from most other substances composed of it, and again let loose the caloric imposed.

Vegetable and animal substances, deprived of life, are decomdecomposed by a slight abstraction or excess, their other component parts being less fixed or more volatile; but, when they are not deprived of life, they are not so easily destroyed; having the power, when alive, in some measure of resisting decomposition.

In them also a much less excess or abstraction than is required for decomposition is productive of disease; and, less than this constitutes the healthy quantity.

Fluids are very differently affected; for the most part they are converted into steam or vapor by an excess, and into solids by abstraction; and, as it is by the former of these operations that we propose to decompose inflammation, I shall endeavour to explain the manner in which this power is produced by the action of caloric upon fluids.

From what has already been observed, it appears, that inflammation is a compound property; and that caloric, or rather an excess of caloric, is one of the constituent parts. This position is confirmed by observing, that without such excess, be the other component parts whatever they may, a part cannot be inflamed; that is, the amount of the others is short of inflammation.

Having shewn that an excess of caloric, that is, a quantity greater than what can be allowed within the limits of health, is essential to form inflammation, the reduction of such excess seems, primâ facie a direct and effectual remedy. But speculative reasoning must, at all times, give way to solid facts deduced from practical observations; I shall therefore take notice of what is particularly observable on the evaporation of alcohol from surfaces inflamed by burning or scalding; alleging, that the same applied to active inflammation in general, by whatever cause produced, when it is proper to remove it by resolution, is highly useful; but must first return to the explanation of the manner in which the power of removing inflammation is connected with the evaporation of fluids.

### *The Way in which the Evaporation of Alcohol decomposes or destroys Inflammation.*

Vapour or steam, like inflammation, is a compound; but it is composed only of caloric and a fluid in the atmospheric air, and is the invariable result of the combination of these two agents.

A fluid of a given volatility or chemical attraction to caloric, or in other words, disposed to be converted into steam steam or vapour, will be dissipated more rapidly than another fluid of less volatility or attraction to caloric; for example, water has an attraction to it, and is proportionately evaporable, under certain circumstances, that is, united with a given quantity. Alcohol has a stronger attraction to caloric than water has; therefore, under similar circumstances, is evaporable at a greater rate. Vitriolic ether has a still stronger attraction; and, therefore, under similar circumstances, is evaporable at a greater rate than alcohol; and the same may be said of all other fluids. The rate then of evaporation, cæteris paribus, will be correspondent to the attraction the fluid bears to caloric.

It must also be observed, that the dissipation of caloric will be in a ratable proportion with the rapidity of the evaporation, viz. Water being evaporated from a surface, in a given time, to the quantum one; alcohol, from the same surface, in the same time, to the quantum two; and ether, from the same suface, in the same time, to the quantum four; the exhaustion of caloric will be with water one, with alcohol two, with ether four. The quantity of cold then induced, or what is the same thing, the quantity of caloric dissipated, will be in the ratio of evaporation, corresponding with the volatility, or evaporability of the fluid employed.

Further, if a piece of iron heated to one hundred degrees, will be cooled by the evaporation of water from its surface in four minutes, we may suppose it will be cooled by the evaporation of alcohol in two minutes, and, by the evaporation of ether in one minute. I do not however, wish to be understood as considering this a correct statement, but have introduced it only to explain, by way of example, the effects necessarily produced by employing fluids of different degrees of evaporability, in the subduction of inflammation. It may not be amiss here to observe, that steam is a compound of caloric and a fluid; and if you abstract the caloric, the product of the remainder is not steam.

### *On the particular Effects observable on the Evaporation of Alcohol, from Surfaces injured by Burning or Scalding.*

Pain is considerably abated at the commencement of the process, and in a short time is wholly removed; but on ceasing the evaporation, is apt to return; is again removed by evaporation; and this alternation may be carried on till inflammation is wholly subdued.

The

The precise length of time required for the subduction of inflammation must depend upon many circumstances, which I shall endeavour to point out when I treat on the most eligible method of conducting the process of evaporation.

When there has not been a destruction of the epidermis, though considerable vesication have been produced, there will not need any other application.

When the injured parts have been deprived of their natural covering, there will be required only the application of wax and oil, or any other easy plaist[illegible], that is commonly employed in simple ulceration, after inflammation is thus removed.

If the parts are actually decomposed by the imposition of great excess of caloric, neither the remedy here recommended, nor any other remedy, will contribute to their restoration. But yet, the adjoining parts will be subject to much inflammation, which will require to be moderated; and therefore the process of evaporation will be equally beneficial in this, as in that of simple inflammation.

The conveniency and elegance of the process are some recommendation; the philosophical principles with which its efficacy is connected invite some attention; the immediate relief from pain declares its utility; its power, under all degrees of diseases, to which it ought to be applied being always apportionate, bespeaks its superiority; and, the invariable uniformity of its action decides in favour of its preference.

### *On the Method of conducting the Process of Evaporation.*

As the object of our attention is the decomposition of inflammation by the abstraction of one of its component parts, it is required that an appropriate method, as well as the subject of evaporation, be pointed out.

Alcohol being already proposed as the subject, I wish to give it the best possible chance, by applying it in such a manner as will be most favourable to its evaporation; and, as I cannot make out a better, I shall relate that which I have always hitherto adopted.

In cases of inflammation only, a single piece of linen cloth has been laid over the parts aggrieved, so as to extend in all directions, considerably beyond them; alcohol has then been assiduously poured, or what is better, squeezed from a sponge upon it, so as to keep the whole of the linen cloth continually moist with alcohol.

When, however, there is no exposure from a separation of of the cuticle, it may be evaporated with equal safety and effect from the skin without the interposition of any thing.

When the injured parts have been deprived of their natural covering, a piece of thin bladder, or a plaister of wax and oil, has been laid upon them, well adapted, and then the linen cloth and alcohol, &c.

This precaution is not necessary when the parts are decomposed by great excess of caloric.

Under all circumstances the excess of cold air will contribute much to the efficacy of the process.

As I before observed, the pain is immediately abated; and in a short time, commonly in one hour, wholly removed; it is nevertheless likely to return in a slight degree, but is again promptly relieved by evaporation; and in all cases, and under all circumstances, the recurrence of pain and heat will point out the necessity of again having recourse to it.

Having thus done all that was proposed, namely, decomposed inflammation, the subsequent treatment must be such as is to be preferred, where there is not too much inflammation; and I shall again, once for all, observe, that where there is not occasion to employ remedies for the resolution or dispersion of inflammation, I do not recommend the process of evaporation.

In the gout, rheumatism, ophthalmy, erysipelas, &c. the effects are particularly striking. I have now under my care, a case of violent erysipelas in the face, with both eyes nearly closed up; the alcohol has just been applied, as above directed, and the disease is destroyed.

I must observe, that I have frequently employed vitriolic ether, with the effects that might naturally be expected from so volatile a fluid; but one accident of some moment, and others of less, from the incautious approach of fire, have made me fearful of recommending it; and the more especially as I have always found alcohol fully equal to the end I proposed, and have very commonly chose to lessen its powers, by mixing water with it.

Ether, alcohol, or alcohol and water, may, however, be used with equal safety, by those who have knowledge and care enough to conduct the management of them.

I have now only to add, that, so soon as inflammation is sufficiently repressed, it will be no longer proper to continue the process of evaporation, lest great mischief result from a contrary power to that which produced it, that is, by a too great abstraction of caloric. For it has been already eady shewn, that a certain relative quantity of caloric is essential to health, and a very different relative quantity to disease; and though each of these admits of considerable latitude, there are, nevertheless, limited boundaries, which, if stepped over on either side, that is, too much or too little caloric, decomposition, and consequently, death must follow.

I would, therefore, urge the necessity of desisting from the process of evaporation so soon as inflammation is sufficiently subdued, at all times denoted by the absence of heat and pain, lest too little caloric be left, or what is the same thing, there be a greater abstraction of it than is consistent with health; and, if carried further, too little caloric be left to prevent decomposition, and thus be productive of gangrene.

